# Cost-utility analysis of empagliflozin in heart failure patients with reduced and preserved ejection fraction in China

**DOI:** 10.3389/fphar.2022.1030642

**Published:** 2022-10-28

**Authors:** Yi Tang, Haiqiang Sang

**Affiliations:** Department Cardiology, The First Affiliated Hospital of Zhengzhou University, Zhengzhou, China

**Keywords:** empagliflozin, heart failure with reduced ejection fraction, heart failure with preserved ejection fraction, cost-utility analysis, China

## Abstract

**Objective:** EMPEROR-Reduced and EMPEROR-Preserved studies showed the benefits of empagliflozin along with a reduction in cardiovascular death or hospitalisation for heart failure (HF). Our aim was to evaluate the economics and effectiveness of adding empagliflozin to the standard therapy for HF with reduced ejection fraction (HFrEF) and HF preserved ejection fraction (HFpEF) in China.

**Methods:** A multistate Markov model was constructed to yield the clinical and economic outcomes of adding empagliflozin to the standard therapy for 65-year-old patients with HFrEF and HFpEF. A cost-utility analysis was conducted, mostly derived from the EMPEROR-Reduced study, EMPEROR-Preserved study, and national statistical database. All costs and outcomes were discounted at the rate of 5% per annum. The primary outcomes were total and incremental costs, quality-adjusted life years (QALYs), and incremental cost-effectiveness ratio (ICER). Sensitivity analyses were also performed.

**Results:** In the HFrEF population, the 10-year incremental cost was $827.52 and the 10-year incremental QALY was 0.15 QALYs, resulting in an ICER of $5,612.06/QALY, which was below the WTP of $12,652.5/QALY. In the HFpEF population, compared with the control group, the incremental cost was $1,271.27, and the incremental QALY was 0.11 QALYs, yielding an ICER of 11,312.65 $/QALY, which was also below the WTP of $12,652.5/QALY. In the HFrEF and HFpEF populations, the results of a one-way sensitivity analysis showed that the risk of cardiovascular death in both groups was the most influential parameter. In the HFrEF population, a probability sensitivity analysis (PSA) revealed that when the WTP thresholds were $12,652.5/QALY and $37,957.5/QALY, the probabilities of being cost-effective with empagliflozin as an add-on were 59.4% and 72.6%, respectively. In the HFpEF population, the PSA results revealed that the probabilities of being cost-effective with empagliflozin as an add-on were 53.1% and 72.2%, respectively.

**Conclusion:** Considering that the WTP threshold was $12,652.5/QALY, adding empagliflozin to standard therapy was proven to be a slightly more cost-effective option for the treatment of HFrEF and HFpEF from a Chinese healthcare system perspective, which promoted the rational use of empagliflozin for HF.

## Introduction

Heart failure (HF) is a clinical syndrome in which the cardiac systolic and/or diastolic functions are significantly inadequate, resulting in an inadequate pump function, which is the leading cause of human death and/or hospitalisation, and it has become a serious global public health problem (Hao et al., 2019). Notably, HF is divided into HF with mildly reduced ejection fraction (HFmrEF), HF with reduced ejection fraction (HFrEF), and HF with preserved ejection fraction (HFpEF), which is more prevalent among the elderly. The clinical characteristics of HFmrEF are more similar to those of HFpEF. Based on the high incidence of poor prognosis and lack of clinically proven therapies thus far, HFpEF is considered to be the only and/or largest unmet demand in the cardiovascular (CV) medicine field (Dunlay et al., 2017). Approximately 1%–2% of the global population over 40 years of age suffer from HF; among the population aged 60–70 years, this proportion would add to 10% and continue to increase with the aging population. The prevalence of HF in China is approximately 1.3%, and it has increased by 44% over the past 15 years (Cook et al., 2014; Conrad et al., 2018). Moreover, HF not only lowers the quality of life but also causes a heavy economic burden for patients and their families. It is estimated that the economic burden of HF care in China will increase significantly in the next few decades. Furthermore, HF is an economically burdensome disease. The cost of hospitalisation for HF in China has increased to approximately $26.3 billion, representing an increase of 87% in 10 years (Cook et al., 2014).

Sodium-glucose cotransporter 2 (SGLT2) inhibitors are new therapeutic agents for diabetes mellitus that decrease blood sugar levels by inhibiting the proximal renal tubular SGLT protein family reabsorption of glucose (Chao and Henry, 2010). The EMPA-REG OUTCOME study demonstrated that the CV safety of empagliflozin was related to a 14% reduction in CV outcomes and a 35% decrease in HF associated with hospitalisation in a diabetic population (Zinman et al., 2016). An EMPEROR-Reduced study found that empagliflozin was associated with a 25% reduction in CV death or hospitalisation for HF and a 30% reduction in HF associated with hospitalisation in patients with HFrEF with or without diabetes (Packer et al., 2020). SGLT2 inhibitors were recommended as the first-line drugs for the treatment of HFrEF based on promising evidence from EMPEROR-Reduced and DAPA-HF studies (Colombo et al., 2020; Packer et al., 2020; Mcdonagh et al., 2021). The EMPEROR-Preserved study also provided evidence of empagliflozin related to a 21% reduction in CV death or hospitalisation for HF and a 29% reduction in HF hospitalisation in patients with HFpEF with or without diabetes (Anker et al., 2021). Based on the satisfactory results of the EMPEROR-Preserved study, the United States Food and Drug Administration has approved that empagliflozin could be used to treat HFpEF in February 2022 (Michael O’Riordan, 2022).

Considering the curative effect, economic benefit was also an important factor in medical decision-making. Empagliflozin contributed to a higher cost of HF that significantly limited its promotion. Cost-utility analysis is a useful method to evaluate the value of drugs by quantifying and comparing the cost and effectiveness of different therapeutic strategies. The previous pharmacoeconomic evaluation focussed on HFrEF or HF as a homogenous group and lacked a pharmacoeconomic evaluation of HFpEF (Griffiths et al., 2014; Park et al., 2019). Therefore, we evaluated the cost-utility of empagliflozin in HFrEF and HFpEF from the perspective of healthcare systems in China.

## Materials and methods

### Simulated population

Two simulated cohorts were employed in this study. The first cohort comprised the HFrEF population, whose characteristics were consistent with those of the EMPEROR-Reduced study.^7^ The second cohort was composed of the HFpEF population, whose characteristics were similar to those of the EMPEROR-Preserved study.^10^ The simulated population in the EMPEROR-Reduced study was not completely different from that in the EMPEROR-Preserved study. The major difference from the EMPEROR-Reduced population was that patients with HFrEF were defined by a left ventricular ejection fraction (LVEF) ≤ 40%, while the population with HFmrEF had an LVEF of 40%–50% and those with HFpEF had an LVEF > 50% in the EMPEROR-Preserved study. The empagliflozin group in both hypothetical cohorts comprised patients who received empagliflozin (10 mg daily) as an add-on to the standard therapy for HF. The control group received placebo and standard treatment for HFrEF and HFpEF. The initial age of the simulated patients in the model was 65 years, and the majority of HF cases were reported in the elderly in the real world (Li et al., 2020). A Markov model was created using Microsoft Excel 2010.

### Model structure

**FIGURE 1:**
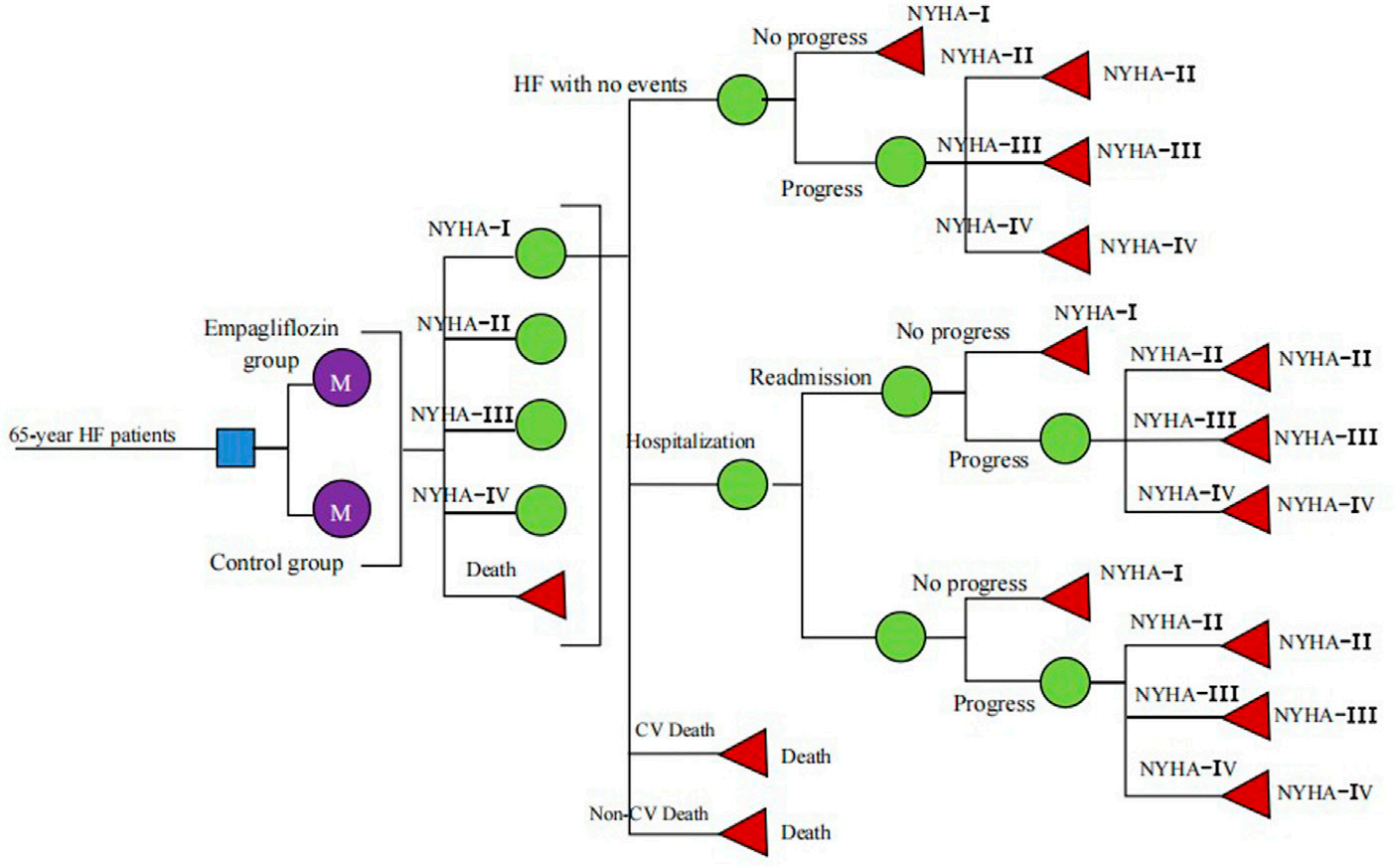
Schematic representation of the Markov model.

Based on the clinical outcomes of the EMPEROR-Reduced and EMPEROR-Preserved studies, including CV death, hospitalisation, and readmission for HF, we constructed a multistate Markov model to evaluate the cost-utility analysis of the intervention (empagliflozin) for patients with HFrEF and HFpEF. We defined five mutually independent and transferable Markov states (Figure 1), including New York Heart Association (NYHA) function classes I, II, III, and IV, and death (CV death and non-CV death). At the end of each cycle, the patient would switch between different NYHA function classes, and the symptoms would improve or worsen. Because the purpose of our study was to calculate the long-term costs and outcomes of HF patients, a lifetime (10 years) horizon with a 3-month cycle length was applied for the cost-utility analyses. The rate of HF readmission was high during the early post-discharge period, especially within the first 90 days after discharge from HF hospitalisation (Khan et al., 2021; Wideqvist et al., 2021). Based on “The Guidelines of Pharmacoeconomic Evaluations of China (2020),” an annual discount rate of 5% was applied to minimise the impact of inflation on future costs and QALYs, and a half-cycle correction was applied to prevent the overestimate of expected survival (Liu, 2020).

### Clinical event probabilities

As we could not directly obtain the age-dependent incidence of each clinical event from the EMPEROR Reduced and EMPEROR Preserved studies, we proposed an assumption that the rate of each clinical event and the efficacy of empagliflozin on HFrEF and HFpEF were fixed. We chose some data from other published literature and national statistical databases in cases where relevant data could not be directly obtained from the EMPEROR-Reduced and EMPEROR-Preserved studies. In the HFrEF population, the risks of CV death were 10.0% and 10.8% in the empagliflozin and control groups, respectively, while the risks of hospitalisation for HF were 13.2% and 18.3% (Packer et al., 2020). The risk of readmission for HF after discharge (13.4%) within 30 days in HFrEF patients was derived from the PARADIGM-HF study (Desai et al., 2016). In the HFpEF population, the incidences of CV death were 8.3% and 7.2% in the empagliflozin and control groups, respectively, and the incidences of HF-related hospitalisation were 16.2% and 12.1%, respectively (Anker et al., 2021; Packer et al., 2021). The incidence of readmission for HF within 30 days after discharge in HFpEF patients was 18% in the I-PRESERVE trial (Carson et al., 2015). Considering that there was no difference in age-dependent non-CV death between both groups, it was assumed that the non-CV death of both groups was the same, as obtained from the China Center for Disease Control and Prevention (National Center for Chronic and Noncommunicable Disease Control and Prevention, 2019). The formula r = −1/t ln(S), P = 1-e^(−r*T) was applied to calculate the event probabilities (S is the rate, t is the time, and P is the clinical event probability) (Park et al., 2019) (Table 1). The transition probabilities between different NYHA function classes at the end of every cycle were assumed to be similar. The 3-month transition probability between NYHA function classes was obtained from the published literature (King et al., 2016) (Table 2).

**TABLE 1:** Selected model inputs.

Variables	Value	Range	Distribution	Reference
Clinical event probabilities				
HFrEF population				
Cardiovascular death				
Control group	0.0212	0.01908–0.02332	Beta	Packer et al. (2020)
Empagliflozin group	0.01956	0.01760–0.02152	Beta	Packer et al. (2020)
Hospitalization for heart faliure				
Control group	0.03719	0.03347–0.04091	Beta	Packer et al. (2020)
Empagliflozin group	0.02619	0.02357–0.02881	Beta	Packer et al. (2020)
Readmission for heat faliure	0.331	0.2979–0.3641	Beta	Desai et al. (2016)
HFpEF population				
Cardiovascular death				
Control group	0.00975	0.00877–0.01072	Beta	Anker et al. (2021)
Empagliflozin group	0.00864	0.00778–0.00951	Beta	Anker et al. (2021)
Hospitalization for heart faliure				
Control group	0.02003	0.01803–0.02204	Beta	Packer et al. (2021)
Empagliflozin group	0.01466	0.01319–0.01613	Beta	Packer et al. (2021)
Readmission for heat faliure	0.417	0.3753–0.4587	Beta	Carson et al. (2015)
Probability of non-CV mortality by age				
65–69 years	0.2430%			(National Center for Chronic and Noncommunicable Disease Control and Prevention, 2019)
70–74 years	0.3042%			(National Center for Chronic and Noncommunicable Disease Control and Prevention, 2019)
75–79 years	0.4185%			(National Center for Chronic and Noncommunicable Disease Control and Prevention, 2019)
Utility (HFrEF)				
NYHA I	1.000	0.950–1.000	Beta	Di Tanna et al. (2021)
NYHA II	0.860	0.817–0.903	Beta	Di Tanna et al. (2021)
NYHA III	0.600	0.570–0.630	Beta	Di Tanna et al. (2021)
NYHA IV	0.280	0.266–0.294	Beta	Di Tanna et al. (2021)
Utility (HFpEF)				
NYHA I	1.000	0.950–1.000	Beta	Di Tanna et al. (2021)
NYHA II	0.830	0.789–0.872	Beta	Di Tanna et al. (2021)
NYHA III	0.550	0.523–0.578	Beta	Di Tanna et al. (2021)
NYHA IV	0.270	0.257–0.284	Beta	Di Tanna et al. (2021)
Hospitalization and readmission	−0.1	−0.13–-0.08	Beta	King et al. (2016)
Cost				
Standard therapy	$131.96	$131.96–310.83	Gammma	Huang et al. (2017)
Empagliflozin	$59.63	$47.70–71.55	Gammma	Local data
Hospitalization and readmission	$1,783.39	$1,029.73–3,336.39	Gammma	Ma, (2021)
Discounted rate	5%	0%–8%		Liu (2020)

**TABLE 2:** New York Heart Association classes transition probabilities per cycle (3 months).

To	I	II	III	IV	Distribution
From					
I	0.977	0.019	0.004	0	Dirichlet
II	0.008	0.981	0.010	0.001	Dirichlet
III	0	0.034	0.960	0.006	Dirichlet
IV	0	0	0.055	0.945	Dirichlet

### Cost and utility

From a Chinese healthcare system perspective, we only enrolled the direct medical costs because they could be measured easily and objectively. The cost in the study included the costs of hospitalisation for HF, standard therapy, and empagliflozin. The cost of hospitalisation for HF, including medical, operation, examination, inspection, berth, administration fees, and medical staff, were obtained from the China Health Statistics Yearbook 2021 (Ma, 2021). Standard therapy consisted of sacubitril/valsartan (SAC/VAL), angiotensin-converting enzyme inhibitors (ACEI), angiotensin receptor blockers (ARB), beta blockers, and spironolactone. Although HFpEF therapy lacked specific drugs, most were already treated with diuretics, SAC/VAL, ACEI, ARB, beta-blockers, and spironolactone. The standard therapy cost was derived from the National Claims Sampling Database (Huang et al., 2017). According to the latest national negotiation price in 2022, empagliflozin was $0.6625 per 10 mg, while SAC/VAL was $0.497 per 100 mg; thus, we could calculate the range of standard therapy and the cost of empagliflozin of each cycle (Table 1). All costs of this study were converted into US dollars at an exchange rate of 1 $ = 6.4 RMB (The People’s Bank of China, 2021).

Owing to the simultaneous lack of evidence on the health utility of HFrEF and HFpEF in China, the published literature was chosen. The visual analogue scale and time trade-off were used to calculate the utility scores of HFrEF and HFpEF (Di Tanna et al., 2021). Additionally, a higher rate of hospitalisations leads to a greater utility decrease; thus, each HF-related hospitalisation would reduce the utility by 0.1 (King et al., 2016) (Table 1). The expenses and QALYs in the model were inflated to 2022 by adopting the consumer price index in the medical care category.

### Outcome

The primary endpoints in this study were the total discounted cost, total discounted QALYs, and ICER. The ICER was calculated by dividing the difference in total cost by the difference in outcomes for both groups. In view of “The Guidelines of Pharmacoeconomic Evaluations of China (2020)”: ICER < 1 fold of gross domestic product (GDP) per capita, the incremental cost was totally deserved and very cost-effective; 1 fold of GDP per capita < ICER < 3-fold of GDP per capita, the incremental cost was receivable and cost-effective; ICER > 3-fold of GDP per capita, the incremental cost was not deserved and not cost-effective (Liu, 2020). Considering that there was no fixed willingness-to-pay (WTP) to evaluate cost utility in China, we defined the WTP thresholds of $12,652.5/QALY and $37,687.5/QALY related to the one-time and three-times GDP per capita of China in 2021, respectively, to judge whether adding empagliflozin in HFrEF and HFpEF was very cost-effective (ICER ≤ $12,652.5) or only a cost-effective (i.e., ICER ≤ $37,687.5) (Eichler et al., 2004).

### Sensitivity analyses

A series of sensitivity analyses, including one-way sensitivity analysis and probability sensitivity analysis (PSA), was adopted in this study to validate the stability of the model. One-way sensitivity analysis was used to calculate all ICERs by changing the reasonable range of one parameter and keeping the other parameters fixed in the model to evaluate the impact of this parameter on cost utility (Table 1). The results are presented in a tornado diagram. Notably, PSA can simultaneously consider the impact of changes of multi-parameters in the model on the ICER. The parameters in the model were randomly sampled (1,000 repetitions) based on their relevant distributions. The cost parameters suited the gamma distribution, while the utility parameters and event probability parameters suited the beta distribution, and the results were represented by cost-effectiveness-acceptability curves and scatter diagrams.

Scenario analysis was also performed by changing the cost of empagliflozin (national purchase price, $0.275 per 10 mg, once daily), the time horizon (15 and 20 years), and the hospital level [town-level hospitals ($1,029.73), county-level hospitals ($1,231.06), municipal hospitals ($1,783.39), provincial hospitals ($1,949.55), and ministerial hospitals ($3,336.39)] (Ma, 2021).

## Results

### HFrEF population

The 10-year total cost in the empagliflozin group was higher than that in the control group ($5,501.48 vs. $4,673.96), projecting an incremental cost of $827.52. However, the 10-year total QALYs in the empagliflozin group was higher than that in the control group (4.27 QALYs vs. 4.12 QALYs), thereby projecting an incremental QALY of 0.15 QALYs. This yielded an ICER of $5,612.06/QALY, which was below the WTP of $12,652.5/QALY. Empagliflozin was associated with a 1.2% reduction in CV death and a 28.7% decrease in HF hospitalisation from our simulated results (Table 3).

**TABLE 3:** The results from base-case analysis.

	Total cost ($)	Total life years (QALY)	Incremental cost ($)	Incremental life years (QALY)	ICER ($ per QALY)
HFrEF population					
Empagliflozin group	5,501.48	4.27	827.52	0.15	5,612.06
Control group	4,673.96	4.12			
HFpEF population					
Empagliflozin group	5,916.20	4.96	1,271.27	0.11	11,312.65
Control group	4,645.23	4.85			

### HFpEF population

Compared with the control group, the 10-year total cost in the empagliflozin group was more expensive ($5,916.20 vs. $4,645.23) and an incremental cost of $1,271.27. However, the 10-year total QALYs in the empagliflozin group was higher (4.96 QALYs vs. 4.85 QALYs), along with an incremental QALY of 0.11 QALYs. This yielded an ICER of $11,312.65/QALY, which was below the WTP of $12,652.5/QALY. Empagliflozin was associated with a 7.8% reduction in CV death and a 25.5% decrease in HF hospitalisation based on our simulated results (Table 3).

### Sensitivity analyses

**FIGURE 2:**
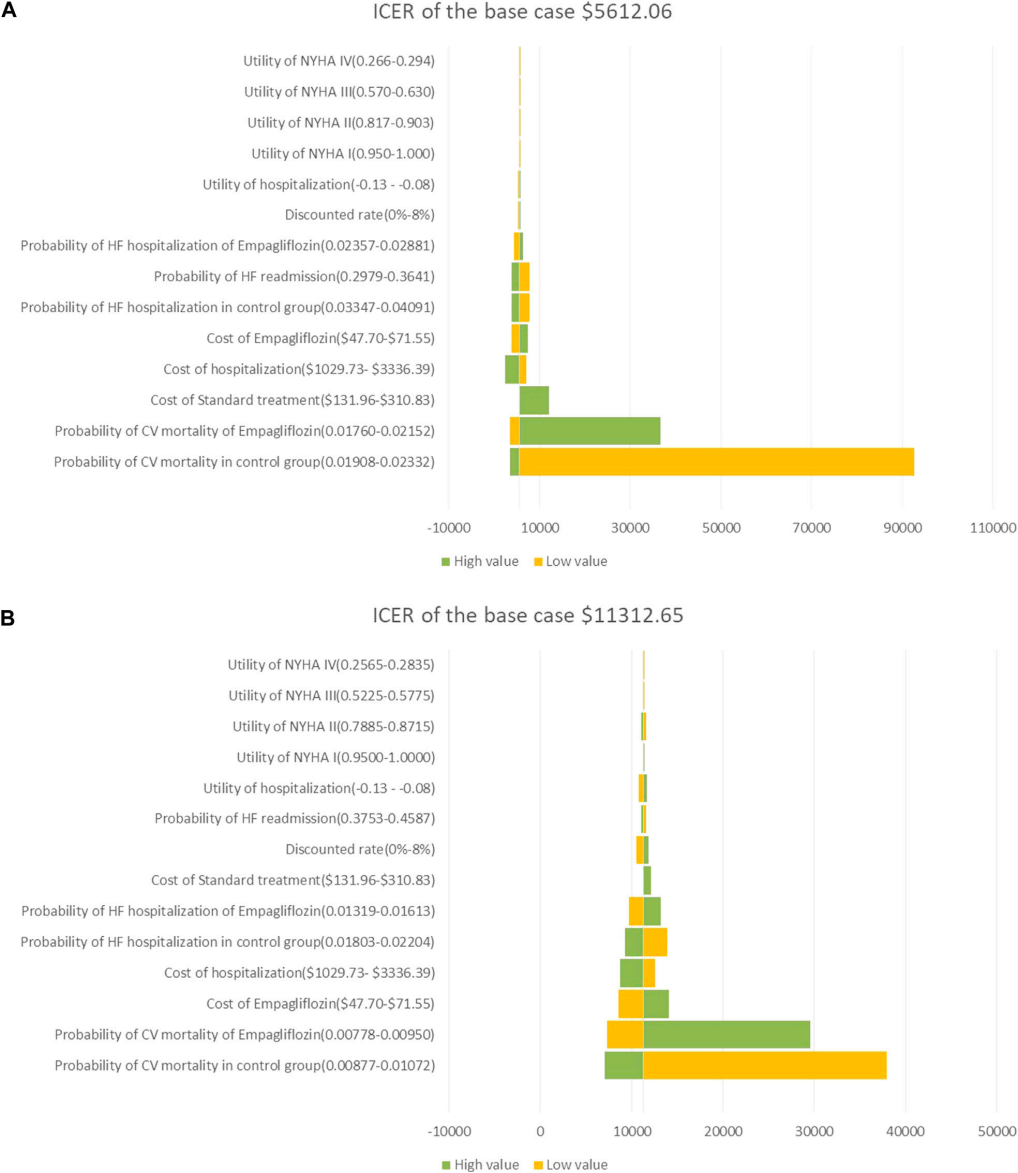
**(A)** Tornado diagram showing the univariate sensitivity analysis of the Markov model simulation in HFrEF population. **(B)** Tornado diagram showing the univariate sensitivity analysis of the Markov model simulation in HFpEF population.

In the HFrEF and HFpEF populations, the results of the one-way sensitivity analysis showed that the risk of CV death in both groups was the most influential parameter, which was more than three times the GDP of $37,957.5/QALY; followed by the cost of empagliflozin and the cost of hospitalization for HF, which was lower than three times the GDP of $37,957.5/QALY; and the ICER calculated by changing a reasonable range of parameters was represented as a tornado diagram (Figures 2A,B).

**FIGURE 3:**
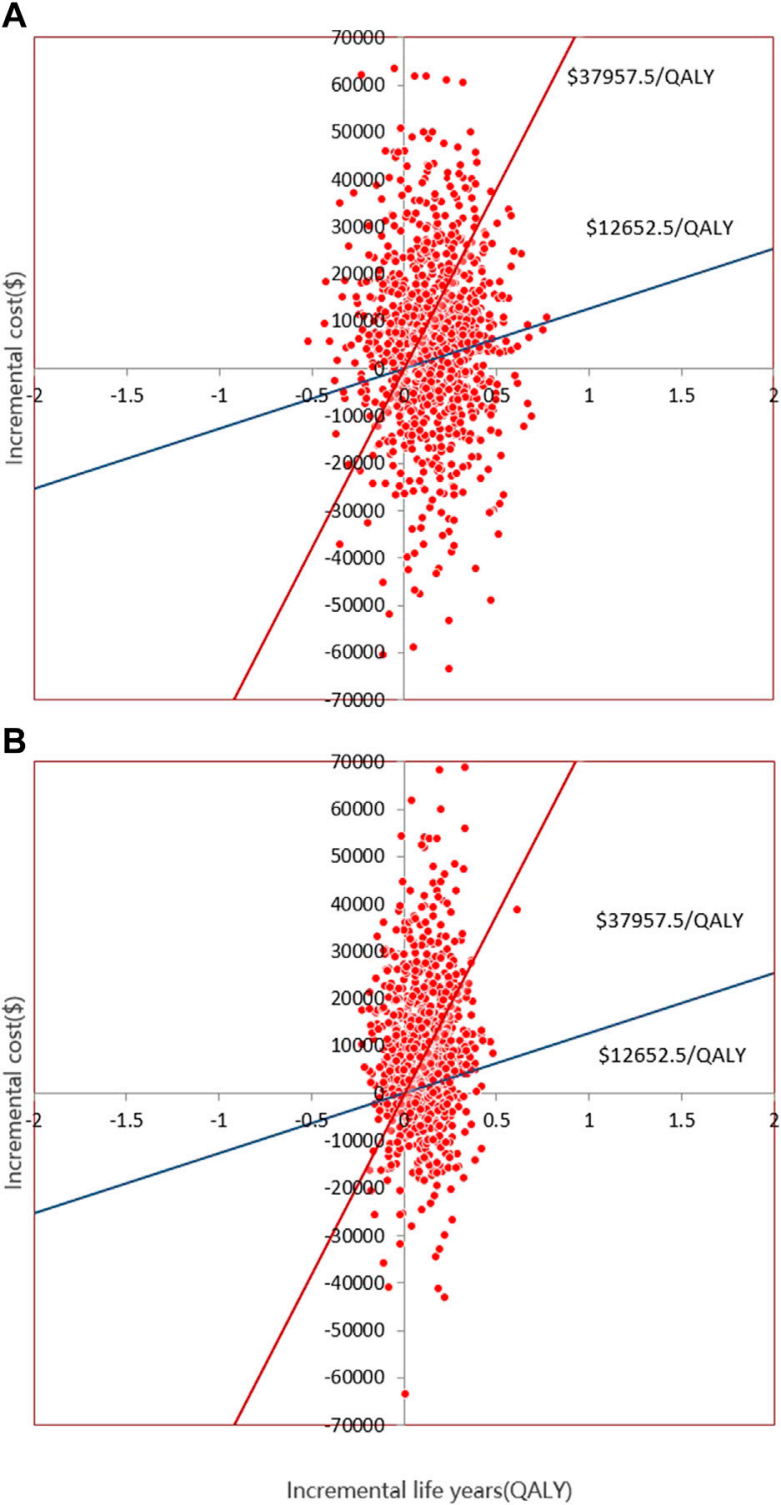
**(A)** Scatter plot showing the incremental costs and incremental quality-adjusted life-year of a thousand simulations for Empagliflozin group and Control group in HFrEF population. **(B)** Scatter plot showing the incremental costs and incremental qualityadjusted life-year of a thousand simulations for Empagliflozin group and Control group in HFpEF population.

**FIGURE 4:**
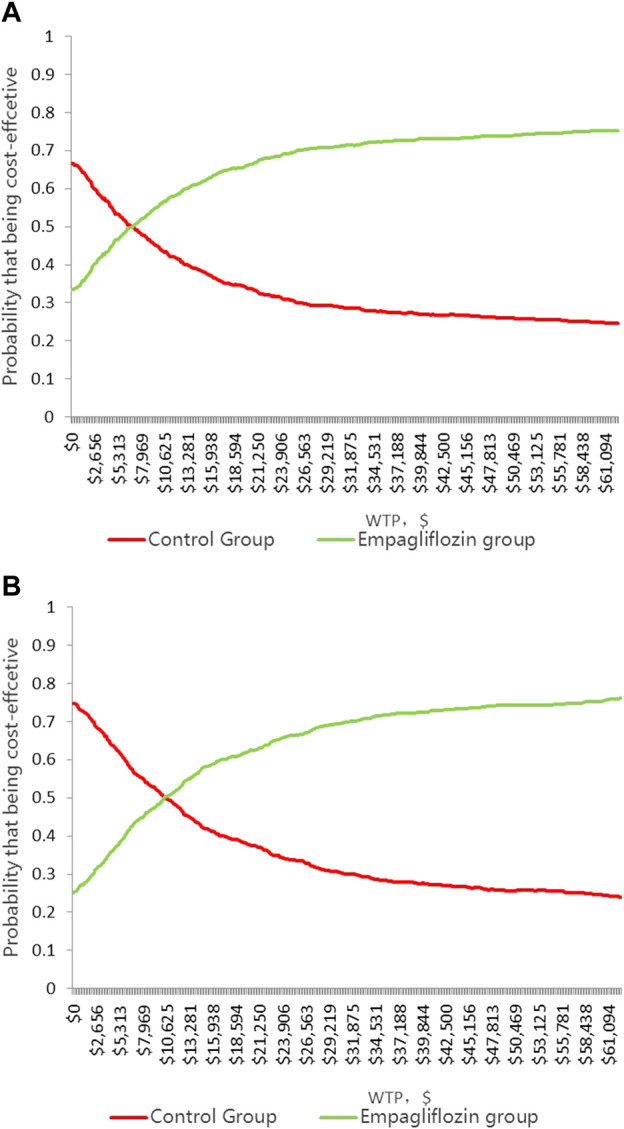
**(A)**Cost-effectiveness acceptability curve showing the maximum willingness to pay and the corresponding probability of cost-effectiveness for Empagliflozin group and Control group in HFrEF population. **(B)** Cost-effectiveness acceptability curve showing the maximum willingness to pay and the corresponding probability of cost-effectiveness for Empagliflozin group and Control group in HFpEF population.

In the HFrEF and HFpEF populations, most of the 1,000 iterations fell in the upper-right quadrant, demonstrating that the add-on empagliflozin treatment for HFrEF and HFpEF usually incurred a higher cost but gained higher QALYs (Figures 3A,B). In the HFrEF population, the PSA results revealed that when the WTP thresholds were $12,652.5/QALY and $37,957.5/QALY, the probabilities of being cost-effective for the add-on empagliflozin treatment were 59.4% and 72.6%, respectively (Figure 4A). In the HFpEF population, the PSA results revealed that when the WTP thresholds were $12,652.5/QALY and $37,957.5/QALY, the probabilities of being cost-effective for the add-on empagliflozin were 53.1% and 72.2%, respectively (Figure 4B).

Based on the scenario analysis, when the national purchase price of empagliflozin was slightly lower, the cost of hospitalisation for HF was much higher, the time horizon was longer, and it was more cost-effective to add empagliflozin to the HFrEF and HFpEF populations (Table 4).

**TABLE 4:** The result of scenario analyses presented as ICER.

Scenario	ICER($ per QALY)	
	HFrEF population	HFrEF population
Price for empagliflozin		
National negotiation price	5,612.06	11,312.65
National purchase price	520.36	3,298.60
Hospital Level		
Town Hospital	7,145.36	1,257.86
County Hospital	6,735.76	12,235.49
Municipal Hospital	5,612.06	11,312.65
Provincial Hospital	5,274.02	11,035.04
Ministerial Hospital	2,452.50	8,717.90
Time horizon		
10 years	5,612.06	11,312.65
15 years	5,147.52	9,842.18
20 years	4,882.05	8,907.66

## Discussion

This study aimed to explore the cost utility of an intervention (empagliflozin) on HFrEF and HFpEF populations based on the EMPEROR-Reduced study, EMPEROR-Preserved study, and national statistical database. In our study, we found that adding empagliflozin to the standard therapy for HFrEF was cost-effective from a Chinese healthcare system perspective with an ICER of 5,612.06 $/QALY, which was below the WTP of $12,652.5/QALY. Adding empagliflozin to the standard therapy for HFpEF generated advantages in cost utility. One HFpEF patient gained a QALY by spending $11,312.65, which was also below the WTP of $12,652.5/QALY. A series of sensitivity analysis was conducted to validate the stability of the model. Generally, the results in the model provided decision-makers and healthcare payers with a valuable quantitative assessment of empagliflozin in the HFrEF and HFpEF populations.

Initial clinical trials showed that SGLT2 inhibitors were very promising antidiabetic drugs but reduced CV death and hospitalisation for HF in the diabetic population (Zinman et al., 2016; Wiviott et al., 2019). The EMPEROR-Reduced and DAPA-HF studies demonstrated that SGLT2 inhibitors benefited HFrEF along with a reduction in CV death and/or hospitalisation for HF in both diabetic and non-diabetic patients (Colombo et al., 2020; Packer et al., 2020). In HFrEF, empagliflozin induced reverse remodeling with regression in LV dilatation, improved exercise capacity and enhanced quality of life (Santos-Gallego et al., 2021; Requena-Ibanez et al., 2022). This was due to empagliflozin causing a shift in myocardial metabolism away from glucose utilization towards consumption of free fatty acids and ketone bodies (Garcia-Ropero et al., 2019; Santos-Gallego et al., 2019; Santos-Gallego et al., 2022). In HFpEF, empagliflozin improved diastolic dysfunction and myocardial fibrosis that caused HFpEF; moreover, empagliflozin also improved other mechanisms contributing to HFpEF such as renal dysfunction and vascular stiffness/systemic vascular resistance (Requena-Ibanez et al., 2021; Santos-Gallego et al., 2021; Santos-Gallego and Van Spall, 2021). A series of studies have proven that adding dapagliflozin to the standard therapy of HFrEF generated cost-effectiveness advantages in different economic systems and medical environments, including the United States, Australia, Thailand, and China (Mcewan et al., 2020; Jiang et al., 2021; Krittayaphong and Permsuwan, 2021). Although the study reported a cost-utility analysis of empagliflozin in patients with chronic HF from the healthcare system perspectives of the United States and the United Kingdom, all the parameters were derived from subgroup data from the EMPA-REG OUTCOME trial, which only included the diabetic population. Therefore, this study might not fully demonstrate the advantages of empagliflozin in HFrEF (Reifsnider et al., 2020). The add-on empagliflozin treatment for HFrEF was a cost-effective choice in Thailand, and it also considered adverse reaction events, including urinary tract infections (Krittayaphong and Permsuwan, 2022). Empagliflozin in HFrEF also generated advantages in cost-effectiveness in the total, diabetic, and non-diabetic populations in China (Lin et al., 2022).

Owing to the lack of specific drugs for HFpEF therapy, SAC/VAL, ACEI, ARB, and spironolactone were unable to reduce the risk of CV or hospitalisation for HF (Yusuf et al., 2003; Massie et al., 2008; Hegde et al., 2017). Empagliflozin was the first to provide promising evidence towards improving HFpEF (Anker et al., 2021). There was little evidence of the cost-utility analysis of empagliflozin in the treatment of HFpEF. A study in Thailand showed that one HFpEF patient gained a QALY by spending $11,809, which was not worthwhile, as the WTP was lower (4,773.27 $/QALY) (Krittayaphong and Permsuwan, 2022). Additionally, several studies have shown that empagliflozin is cost-effective in the treatment of diabetes (Ramos et al., 2020); therefore, adding empagliflozin to standard therapy was a duly cost-effective choice for HFrEF and HFpEF patients with diabetes.

There were several reasons why empagliflozin was more cost-effective in HFrEF than in HFpEF. First, the data in the model were mostly based on the EMPEROR-Reduced and EMPEROR-Preserved studies (Packer et al., 2020; Anker et al., 2021). Empagliflozin could reduce more hospitalisation times for HFrEF than HFpEF, which reduced hospitalisation costs and increased QALYs. The hazard ratio (0.69, 95% CI, 0.59–0.81) of hospitalisation for HF in the EMPEROR-Reduced study was lower than that in the EMPEROR-Preserved study (0.72, 95% CI, 0.63–0.82) (Packer et al., 2020; Anker et al., 2021). Second, the severity of HFrEF was higher than that of HFpEF in the EMPEROR-Preserved study, and the EMPEROR-Reduced study showed a higher mortality rate for HFrEF.

In the one-way sensitivity analysis, CV death in both groups was the most important driver in cost utility, regardless of HFrEF or HFpEF population, which had more than three times the GDP of $37,957.5/QALY, and other parameters had little impact on the model. This finding was expected in our study because empagliflozin could not reduce the risk of CV death in the HFrEF and HFpEF populations from the EMPEROR-Reduced and EMPEROR-Preserved studies (Packer et al., 2020; Anker et al., 2021). If the parameter range was slightly changed, the ICER would change significantly, which could not be attributed to model instability. To determine the cost-utility of the add-on empagliflozin treatment for HFrEF and HFpEF population was a reduction of CV death and/or hospitalization for HF, which also urged us to explore the cost-utility of adding empagliflozin to standard therapy in HFrEF and HFpEF population.

This study has some limitations that should be discussed. First, we derived clinical event probabilities based on the median follow-up times and carried fixed transitional probabilities forward, which might not reflect the real HF course, but the sensitivity analysis validated that our model was stable over a relatively wide range of parameters. Second, hospitalisation for non-HF which was complicated in the real world, was not enrolled in our model, but the EMPEROR-Reduced and EMPEROR-Preserved studies showed that empagliflozin could also reduce the risk of all-cause hospitalisation by 15% and 7%, respectively (Packer et al., 2020; Anker et al., 2021). Our results may be more cost-effective when considering the condition. Third, data on clinical events and utility came from other sources, which might cause racial bias, but we solved the problem that might have occurred *via* sensitivity analysis. Fourth, we assumed that HF patients in the model could tolerate the recommended dose without adverse reaction events, but the EMPEROR-Reduced and EMPEROR-Preserved studies showed that the most common adverse reaction events, including urinary tract infection and hypovolaemia, were not significantly different. Finally, this was a mathematical model combined with national conditions in China, and the generalisability of our findings should be limited to settings or contexts similar to those of this study.

## Conclusion

At a WTP level of $12,652.5/QALY, empagliflozin was proven to be a cost-effective add-on therapy for both HFrEF and HFpEF, from a Chinese healthcare system perspective. The results may serve as a reference for rational drug use and health decision-making, but further cost-utility analyses based on real-world evidence of populations in China need to be performed. Liu, 2020, National Bureau, 2019, Santos-Gallego et al., 2021, The People’s Bank of China, 2020.

## Data Availability

The original contributions presented in the study are included in the article/supplementary material, further inquiries can be directed to the corresponding author.
